# MicroRNA binding site variants–new potential markers of primary osteoporosis in men and women

**DOI:** 10.3389/fgene.2024.1470310

**Published:** 2024-10-01

**Authors:** Bulat Yalaev, Roman Deev, Anton Tyurin, Ramil Salakhov, Kirill Smirnov, Anna Eremkina, Natalia Mokrysheva, Ildar Minniakhmetov, Rita Khusainova

**Affiliations:** ^1^ Laboratory of Genomic Medicine, Endocrinology Research Centre, Moscow, Russia; ^2^ Internal Medicine Department, Bashkir State Medical University, Ufa, Russia

**Keywords:** osteoporosis, fractures, microRNA binding sites, femoral neck, spine, radial bone

## Abstract

**Introduction:**

The identification of significant DNA markers of primary osteoporosis may gain new insights by studying genome regions involved in mechanisms of epigenetic regulation through interactions with microRNAs.

**Methods:**

The authors searched for associations of polymorphic variants of microRNA binding sites of mRNA target genes and polymorphic loci of microRNA genes with primary osteoporosis in a cohort of women and men from the Volga-Ural region of Russia (N = 1.177).

**Results:**

Using case-control association analysis, the authors found that rs1061947 (*COL1A1*), rs10793442 (*ZNF239*), rs6854081 (*FGF2*), and rs11614913 (*miR-196a*) were associated with osteoporotic fractures; rs5854 (*MMP1*) and rs2910164 (*miR-146a*) were associated with low bone mineral density; and rs10098470 (*TPD52*), rs11540149 (*VDR*), rs1042673 (*SOX9*), rs1054204 (*SPARC*), and rs1712 (*FBXO5*) were markers of both fractures and low bone mineral density. Among the identified associations, ethno specific trends were found, as well as sex-specific associations. Prognostic models were developed, among which the model for predicting osteoporosis in general in women (Area Under Curve = 0.909) achieved the highest level of predictive value. Thus, the potential role of polymorphic variants of microRNA binding sites in the development of primary osteoporosis in men and women from the Volga-Ural region of Russia was demonstrated.

## 1 Introduction

Osteoporosis (OP) is an age-dependent metabolic bone disease characterized by a decrease in bone mineral density (BMD) and destruction of bone microstructure, which can lead to elevated bone fragility and fractures (Pisani et al., 2016). There are approximately 18.3% with OP worldwide (Salari et al., 2021). The aging of the population increases the disease prevalence, so treating OP becomes a major financial issue. The disease is primarily associated with postmenopausal women. However, approximately 25% of OP cases occur in men over 50 years of age. Its incidence is thought to be underestimated because men are under-screened for OP compared to women (Salari et al., 2021), this results in men having more complications of the disease such as vertebral or hip fractures, and they have a higher mortality rate from osteoporotic fractures compared to women (Rinonapoli et al., 2021; Wright et al., 2014). In Russia, OP is diagnosed in about 34% of women and 27% of men when a densitometric examination is performed on a random sampling (Zaigrova et al., 2017; Sozen et al., 2017; Lesnyak et al., 2018). Osteoporosis is characterized by increased bone resorption, prevailing over mineralization and anabolic processes, which leads to impaired bone strength and elevated risk of fractures, even from minor falls or impacts (Foger-Samwald et al., 2020). Genomic and multi-omics projects showed that primary osteoporosis is strongly associated with hereditary factors (Makitie et al., 2019), which determine up to 65% of the variability in bone mineral density levels (Howard et al., 1998).

At the same time, quite a few DNA loci with a high risk effect were identified (Rivadeneira et al., 2009). A meta-analysis of a genome-wide association search (GWAS) identified about 56 loci correlating with low BMD and 14 loci associated with fracture risk (Estrada et al., 2012). Morris et al. (2019) identified more than 1,000 conditionally independent signals at genome-wide significance (*p* < 6.6 × 10^−9^) mapping to 515 loci. However, replication of GEFOS/GENOMOS results on an independent sampling from the Volga-Ural region of Russia (VUR) did not confirm most of the identified DNA markers (Estrada et al., 2012). VUR is an ethnically heterogeneous region on the border of Europe and Asia, separated by the Ural Mountains. Turkic, Finno-Ugric, and Slavic peoples live here, having in their gene pool European and Mongoloid components in different proportions (Khusnutdinova et al., 1999).

Aberrations of epigenetic mechanisms are significantly correlated with elevated fracture risk and decreased BMD (Khusnutdinova et al., 1999; Xu et al., 2021). This is supported by studies of DNA methylation patterns (Visconti et al., 2021) and the microRNA pool in blood or bone tissue in patients with OP (Huai et al., 2020). Non-coding RNAs (ncRNAs) act synergistically to regulate cell proliferation, osteoclastogenesis, osteogenesis, autophagy, and other processes. MicroRNA and long ncRNA control multiple signaling pathways and gene expression. Therefore, understanding how binding site polymorphism affects the effect of RNA interference expands the possibilities for developing targeted therapies tailored to the affinity of microRNAs for a specific mRNA target when delivering RNA vectors (Lin et al., 2021). As a therapeutic precedent, gene therapy for musculoskeletal diseases has been well demonstrated in a murine ovariectomy model over the past 2 decades (Baltzer et al., 2001). The development of RNA therapies for parandontal remodeling through the regulation of microRNA activity affecting orthodontic tooth movement seems very promising (Chen and Zhang, 2023). Thus, microRNAs are promising markers for the development of diagnostic strategies and targeted therapy for primary OP (Wang and Grainger, 2012). However, there is a knowledge gap regarding the role of microRNA binding sites in forming the risk of primary OP.

Because each mRNA differs in its ability to bind to microRNAs, some do so more efficiently than others, and this is associated with sequence variations in the 3′-UTR untranslated regions of mature mRNAs (Plotnikova et al., 2019). In addition, different allelic variants have an affinity for different types of microRNAs, altering the qualitative composition and effect(s) of DNA-RNA interactions (Cloonan, 2015). Thus, polymorphism of the 3′-UTR sequence of mRNA changes the binding affinity to microRNAs and thereby may alter the regulation of target mRNA translation or induce mRNA degradation, leading to differential expression of target genes (Zhang and Wang, 2017). Previously, using the method of polygenic risk assessment, the authors developed models predicting the risk of fracture development in women from the Volga-Ural region of Russia, which, along with other loci, included polymorphic variants of microRNA binding sites (Yalaev et al., 2022). The first results in this direction were obtained by Lei et al.(2011) in a full genomic study of microRNA binding sites, where statistically significant associations of 7 polymorphic variants–rs6854081, rs1712, rs10518716, rs17054320, rs10793442, rs10098470, and rs2745426 with femoral neck BMD were revealed. The most significant associations with OP were described for rs1712 of the *FBX05* gene and rs6854081 of the *FGF2* gene (Lei et al., 2011). Another study found associations between rs735890 and lumbar low BMD in the elderly, probably due to changes in microRNA target sites (Amjadi-Moheb et al., 2018). Ahn et al. (2020) investigated microRNA binding site polymorphic variants in mRNA of genes involved in vitamin B metabolism, finding that combinations of risk alleles of the rs9426 (*CD320*), rs10418 (*TCN2*), rs1051296 (*SLC19A1*), and rs16862199 (*SLC19A2*) loci were associated with postmenopausal OP and compression fractures in women of Korean ancestry. Thus, studies show a significant role of polymorphic variants of microRNA binding sites in elevating the risk of osteoporotic fractures and decreasing BMD. However, studies in this direction are sporadic and need to expand the number of studied genes. In addition, replication of the previously obtained results of some authors on different cohorts of patients with primary OP from the other populations and ethnically differentiated groups is no less relevant.

The aim of this study was to search for associations of polymorphic variants of microRNA binding sites in the mRNA of genes involved in the metabolism of connective tissue in general and bone tissue in particular: rs1061947 (*COL1A1*), rs1031820 (*COL11A1*), rs9659030 (*COL11A1*), rs11540149 (*VDR*), rs6854081 (*FGF2*), rs1042673 (*SOX9*), rs10793442 (*ZNF239*), rs10098470 (*TPD52*), rs1054204 (*SPARC*), rs1712 (*FBXO5*), and rs5854 (*MMP1*), as well as associations of polymorphic variants rs2910164 and rs11614913 of the *miR-146a* and *miR-196a* genes with primary OP and development of clinical and genetic models. It was done to predict the risk of fractures and low BMD in general and standard localizations in men and women from the Volga-Ural region of Russia.

## 2 Materials and methods

### 2.1 Phenotypic information

The case-control study included 701 postmenopausal women (mean age = 61.95 ± 7.94) and 501 men (mean age 62 ± 10.8) who underwent medical examination between 2004 and 2011 at the City Clinical Hospitals No. 5, No. 21, and No. 22 in Ufa and Regional Clinical Hospital No. 1. in Yekaterinburg. Ethnic composition of women: Russians–516, Tatars–185. Ethnic composition of men: Russians–470, Tatars–31. The sampling of patients consisted of people with primary OP; the control group included people without fractures and with a normal level of BMD. In the group of women, the number of patients with fractures in general is 280, with fractures in typical localization–160, with low BMD–324. In the group of men, the number of patients with fractures in general is 145, with fractures in typical localization–83, with low BMD–304. The subgroups and their detailed characteristics are summarized in Table 1. The authors completely excluded the presence of any family groups and relatives on the basis of questionnaires and family history data. Exclusion criteria included a history of alcohol and drug abuse, long-term use of glucocorticoids, hormone replacement therapy, current treatment of acute diseases, and the presence of chronic diseases that affect bone metabolism. The level of BMD was measured by Dual-energy X-ray absorptiometry (DEXA) using a Hologic QDR 4500/A DXA system (United States) in standard localizations (femoral neck and lumbar spine) [DEXA system in Ufa and Yekaterinburg demonstrated a high level of reproducibility (coefficient of variation CV<2.6%) and a low level of error (ε<1.5%)]. The general sampling was divided according to the T-criterion–a T value between +2.5 and −0.9 standard deviations (SD) characterized normal BMD, values between −1.0 and −2.5 SD–osteopenia, and values less than −2.5–OP (according to the World Health Organization’s recommendations). The presence of osteoporotic fractures in standard localizations (axial part of the femur, lumbar spine) in general and separately, as well as in combination with any other skeletal fractures, was taken into account. Each participant signed an informed consent form for participation in the study in accordance with the standards of the World Medical Association Declaration of Helsinki “Ethical Principles for Scientific Medical Research Involving Human Subjects.”

**TABLE 1:** Characteristics of the patient cohort by phenotypic subgroups.

Woman (N = 701)			
Parameters	N	Age, years, Me±SD	BMI, kg/m^2^, Me±SD
With fractures	280	62.16 ± 7.95	27.00 ± 3.60
Without fractures	421	60.14 ± 8.01	27.80 ± 3.81
With typical fractures	160	62.49 ± 7.90	28.10 ± 5.40
Without typical fractures	447	62.47 ± 7.90	28.50 ± 5.10
With low BMD	324	62.17 ± 7.95	27.04 ± 3.60
With normal BMD	172	60.23 ± 7.97	28.10 ± 4.90
Low BMD of the femoral neck	170	62.51 ± 7.93	28.50 ± 5.30
Low BMD of the lumbar spine	169	62.50 ± 7.92	28.50 ± 5.10
With low BMD and with fractures	247	60.15 ± 7.43	27.00 ± 2.40
With normal BMD and without fracture	239	61.76 ± 7.96	28.90 ± 3.70
Men (N = 501)			
With fractures	145	62.30 ± 10.83	27.54 ± 2.73
Without fractures	356	59.20 ± 9.02	27.59 ± 4.90
With typical fractures	83	60.61 ± 11.95	28.00 ± 4.37
Without typical fractures	354	60.34 ± 12.23	29.00 ± 4.00
With low BMD	304	62.17 ± 6.90	27.04 ± 3.60
With normal BMD	197	61.23 ± 9.01	27.61 ± 4.60
Low BMD of the femoral neck	216	60.32 ± 12.21	29.00 ± 4.31
Low BMD of the lumbar spine	238	60.27 ± 12.17	29.00 ± 4.32
With low BMD and with fractures	116	63.24 ± 9.51	27.48 ± 3.60
With normal BMD and without fracture	168	61.57 ± 6.97	27.82 ± 2.70

### 2.2 Genetic data

DNA was isolated using the phenol-chloroform extraction method from peripheral blood leukocytes according to the protocol of Mathew (1985). The quality of isolated DNA was checked using a NanoDrop 1,000 spectrophotometer (Thermo Scientific, United States). DNA concentration was measured using a Qubit 4 fluorimeter (Thermo Scientific, United States). Genotyping of the studied samples was performed using KASP™ (Kompetitive Allele Specific PCR) technology in “real-time” by the endpoint (Semagn et al., 2013) on the QuantStudio 12K Flex Real-Time platform. Target microRNA loci were selected using the microRNA database (http://compbio.uthsc.edu/miRSNP, access: 1.02.2024) (Table 2).

**TABLE 2:** Characteristics of polymorphic variants of microRNA binding sites in mRNAs.

No.	Polymorphism		Localization	mRNA of the gene
1	rs1061947	c.*744 C>T	17q21.3–3′-UTR region	*COL1A1*
2	rs1031820	c.*105 C>T	1p21.1, 3′- UTR region	*COL11A1*
3	rs9659030	c.*1183 A>G	1p21.1, 3′- UTR region	*COL11A1*
4	rs11540149	c.*1865 G>A	12q13.11, 3′- UTR region	*VDR*
5	rs6854081	c.*3156 T>C	4q28.1, 3′- UTR region	*FGF2*
6	rs1042673	c.*811 A>G	17q24.3, 3′- UTR region	*SOX9*
7	rs5854	c.*269 C>T	11q22.2., 3′- UTR region	*MMP1*
8	rs10793442	c.*332 G>T	10q11.21, 3′- UTR region	*ZNF239*
9	rs10098470	c.*1073 C>T	8q21.13, 3′- UTR region	*TPD52*
10	rs1054204	c.*582 G>C	5q33.1, 3′- UTR region	*SPARC*
11	rs1712	c.*433 C>T	6q25.2, 3′- UTR region	*FBXO5*
12	rs2910164	n. 303 C>G	5q33.3, mature miRNA variant	*miR-146a*
13	rs11614913	n. 78 C>T	12p38.1, mature miRNA variant	*miR-196a*

### 2.3 Statistical analysis

The PLINK software (v. 1.09) was used to search for associations of alleles and genotypes with case-control fractures and a low BMD level using Pearson’s concordance criterion. The degree of association was assessed in odds ratio (OR) values using the formula: OR = (a*d)/(b*c), where a is the frequency of the trait in the sampling of patients, b is the frequency of the trait in the control sampling, c is the sum of the frequencies of the other traits in the sampling of patients, and d is the sum of the frequencies of the other traits in the control sampling. Tests were performed for the two-sided significance level; differences at *p* < 0.05 were considered statistically significant.

The PLINK program (v. 1.09) was used for meta-analysis of the results. To calculate the mean OR and significance level, fixed effects models (Mantel-Haenszel method) and random effects models (Dersimonian-Laird method) were considered. To assess the statistical heterogeneity of different samples, Cochran’s Q test was used; differences at *p* < 0.1 were considered statistically significant. The level of heterogeneity was determined using the statistical criterion I^2^ (the proportion of variability due to the heterogeneity of samplings) (Higgins and Thompson, 2002). When the I^2^ value was less than 30%, heterogeneity was assessed as mild; when I^2^ was between 30% and 50%, it was assessed as moderate; and when I^2^>50%, it was assessed as highly heterogeneous.

Logistic regression analysis was performed using the MedCalc software (v. 22.016). The disease phenotype (presence/absence of fracture, low BMD/normal BMD, combined conditions) served as the dependent variable, and risk alleles of polymorphic variants and clinical features (BMI, BMD level) served as independent variables (predictors). In the course of logistic regression analysis, several dozens of regression equations were obtained, which the equations with the highest values of statistical significance indices were selected from. To determine the quality of the obtained prediction model, ROC (Receiver Operating Characteristic) analysis was used, the sensitivity and specificity of the model were determined, as well as the AUC (Area Under Curve)–an indicator of the area under the ROC curve (Wray et al., 2010).

### 2.4 Bioinformatics analysis

To evaluate the functional significance of polymorphic variants associated with the risky phenotype, the authors *in silico* predicted microRNAs that potentially interact with mRNAs of genes, wherein the studied high-risk loci are localized. For this purpose, we used miRBase, NCBI, and IntaRNA 2.0 bioinformatics tool for prediction of mRNA-microRNA interactions to analyze changes in the qualitative composition of microRNAs depending on allelic variants of SNP that reached a high level of significance within the association analysis. Three files were prepared for this purpose:

1. Original reference sequences of gene mRNA transcripts in FASTA format from NCBI repositories.
2. Altered reference sequences of mRNA transcripts with alternative risk allele in FASTA format from NCBI repositories.
3. Sequences of all human microRNAs from the mirBase database.

Further, IntaRNA computational algorithms and these files were used to predict all microRNAs that had affinity for mRNA transcripts separately for the reference sequence of known transcripts and the sequences of transcripts with an alternative risk allele. After that, we excluded all those microRNAs which minimum free energy of hybridization was >-8 from all microRNAs that were predicted for these transcripts. After filtering, we matched and compared the lists of these microRNAs for the original mRNA transcript sequence and the new transcript sequence with the alternative allele.

## 3 Results of genetic association analysis

Associations were searched using the nonparametric χ^2^ criterion in the total sampling, taking into account ethnicity and sex, as well as the localization of the phenotypic features of OP: fractures and low BMD. The “typical fractures” group included fractures of the femoral neck, spine, and radial bone, and the “general fractures” group included individuals with fractures in any parts of the skeleton without regard to their localization. In addition, analysis was performed taking into account fractures or a low BMD level in individual localizations. The BMD level was categorized according to the T-criterion (low BMD level at −2.5 SD and below, up to −0.9 SD–normal mineral density level). The identified statistically significant associations are presented in Tables 5, 6, full data in supplementary data.

The Hardy-Weinberg equilibrium test revealed some deviation at the rs1712 locus (*p* = 0.014) in the male sampling, while equilibrium was maintained in the control group without fractures (*p* = 0.159). Therefore, all polymorphic loci were eligible for the association study. The minor allele frequency ranged from 8.4% at the rs1031820 locus to 46.8% at the rs1054204 locus (Table 3).

**TABLE 3:** Characterization of the studied polymorphic loci.

SNP	H_obs_	H_pred_	HW_pval_	MAF	Risk allele
Cohort of Women					
rs10793442	0.250	0.233	0.080	0.135	T
rs11540149	0.145	0.179	0.279	0.100	A
rs1061947	0.298	0.291	0.697	0.177	T
rs10098470	0.034	0.034	1.000	0.017	T
rs1042673	0.485	0.474	0.673	0.386	G
rs5854	0.426	0.433	0.739	0.317	A
rs9659030	0.298	0.298	1.000	0.182	G
rs1031820	0.156	0.155	1.000	0.084	G
rs6854081	0.210	0.201	0.355	0.113	C
rs1054204	0.491	0.498	0.755	0.468	C
rs1712	0.207	0.214	0.447	0.122	C
rs11614913	0.509	0.489	0.359	0.425	T
rs2910164	0.319	0.418	0.457	0.297	G
Cohort of Men					
rs10793442	0.235	0.229	0.868	0.132	T
rs11540149	0.159	0.166	0.622	0.091	A
rs1061947	0.275	0.296	0.235	0.181	T
rs10098470	0.058	0.056	1.000	0.029	T
rs1042673	0.510	0.473	0.07	0.401	G
rs5854	0.419	0.429	0.750	0.311	A
rs9659030	0.293	0.275	0.495	0.164	G
rs1031820	0.138	0.421	1.000	0.301	G
rs6854081	0.169	0.173	0.763	0.096	C
rs1054204	0.520	0.498	0.140	0.452	C
rs1712	0.240	0.320	0.014	0.200	C
rs11614913	0.482	0.484	0.852	0.404	T
rs2910164	0.331	0.326	0.923	0.205	G

Note: H_obs_, observed heterozygosity; H_pred_, predicted heterozygosity.

HW_pval_, *p*-value for assessing compliance with Hardy-Weinberg equilibrium.

The authors conducted a sequential analysis of associations with OP in general–fractures in typical localizations and/or low BMD, as well as with general fractures and various localizations without regard to BMD, low BMD in general and various localizations without regard to the presence of fractures in the total sample. Then, it was conducted in cohorts of men and women separately, taking into account their ethnicity. Certain regularities were found: some associations were characterized by an increase in the level of statistical significance in the analysis of associations taking into account the fracture localization and BMD level, as well as ethnicity and sex (Tables 4, 5).

**TABLE 4:** Identified associations by pairwise comparison in the total sampling of men and women.

Allele	Loci ID	Gene	Phenotype	Sex	Ethnos	χ^2^	OR	CI	*p*-value
A	rs10098470	*TPD52*	Osteoporosis	M + F	Total	5.285	2.15	1.103–4.189	0.022
A	rs10098470	*TPD52*	Osteoporosis	M + F	Russians	9.291	4.07	1.534–10.810	0.002
A	rs11540149	*VDR*	Osteoporosis	M + F	Total	11.390	1.67	1.238–2.260	0.001
A	rs11540149	*VDR*	Osteoporosis	M + F	Russians	14.610	1.94	1.375–2.743	1.32e^−04^
T	rs11614913	*miR-196a*	Osteoporosis	M + F	Total	4.757	1.21	1.020–1.446	0.030
T	rs11614913	*miR-196a*	Osteoporosis	M + F	Russians	4.930	1.25	1.026–1.516	0.030
C	rs1712	*FBX05*	General fractures	M + F	Total	4.451	1.34	1.020–1.758	0.035
C	rs1712	*FBX05*	General fractures	M + F	Russians	5.027	1.41	1.044–1.916	0.025
A	rs10098470	*TPD52*	General fractures	M + F	Russians	10.05	3.57	1.545–8.272	0.002
A	rs11540149	*VDR*	General fractures	M + F	Total	5.201	1.42	1.049–1.909	0.023
A	rs11540149	*VDR*	General fractures	M + F	Russians	7.244	1.58	1.130–2.216	0.007
T	rs11614913	*miR-196a*	General fractures	M + F	Total	6.440	1.26	1.055–1.516	0.011
C	rs1712	*FBX05*	Typical fractures	M + F	Russians	5.226	1.54	1.061–2.233	0.022
A	rs10098470	*TPD52*	Typical fractures	M + F	Total	11.870	2.30	1.559–5.765	5.691e^−04^
A	rs10098470	*TPD52*	Typical fractures	M + F	Russians	17.120	5.20	2.186–12.38	3.510e^−05^
A	rs11540149	*VDR*	Typical fractures	M + F	Total	6.849	1.60	1.122–2.273	0.009
A	rs11540149	*VDR*	Typical fractures	M + F	Russians	8.196	1.77	1.193–2.635	0.004
A	rs11540149	*VDR*	Femoral neck fracture	M + F	Russians	9.075	2.68	1.378–5.202	0.003
G	rs6854081	*FGF2*	Femoral neck fracture	M + F	Total	9.001	2.18	1.296–3.678	0.003
G	rs6854081	*FGF2*	Femoral neck fracture	M + F	Russians	9.801	2.45	1.375–4.357	0.002
G	rs1031820	*COL11A1*	Femoral neck fracture	M + F	Total	4.561	2.44	1.048–5.660	0.033
G	rs1031820	*COL11A1*	Femoral neck fracture	M + F	Russians	5.633	3.76	1.164–12.12	0.018
A	rs10098470	*TPD52*	Lumbar fractures	M + F	Total	13.730	4.00	1.816–8.809	2.107e^−04^
A	rs10098470	*TPD52*	Lumbar fractures	M + F	Russians	23.600	7.77	2.948–20.470	1.183e^−06^
A	rs11540149	*VDR*	Lumbar fractures	M + F	Total	9.592	2.02	1.285–3.186	0.002
T	rs1061947	*COL1A1*	Lumbar fractures	M + F	Total	4.968	1.51	1.049–2.174	0.026
G	rs2910164	*miR-146a*	Lumbar fractures	M + F	Total	4.889	1.55	1.048–2.291	0.027
C	rs1712	*FBX05*	Radial fractures	M + F	Russians	4.980	1.74	1.065–2.855	0.026
A	rs10098470	*TPD52*	Radial fractures	M + F	Total	4.248	2.37	1.018–5.513	0.039
A	rs10793442	*ZNF239*	Radial fractures	M + F	Tatars	5.032	2.31	1.094–4.873	0.024
A	rs10098470	*TPD52*	Low BMD in general	M + F	Total	4.534	2.15	1.045–4.423	0.033
A	rs11540149	*VDR*	Low BMD in general	M + F	Total	4.087	1.38	1.009–1.879	0.043
A	rs11540149	*VDR*	Low BMD in general	M + F	Russians	5.477	1.55	1.071–2.237	0.019
A	rs1031820	*COL11A1*	Low BMD in general	M + F	Tatars	3.868	1.84	0.996–3.407	0.049
A	rs10793442	*ZNF239*	Low BMD in general	M + F	Russians	4.052	1.35	1.007–1.818	0.044
A	rs10098470	*TPD52*	Femoral neck low BMD	M + F	Total	5.139	2.38	1.100–5.158	0.023
A	rs10098470	*TPD52*	Femoral neck low BMD	M + F	Russians	6.359	3.78	1.248–11.470	0.011
A	rs1031820	*COL11A1*	Femoral neck low BMD	M + F	Tatars	6.426	2.41	1.202–4.814	0.011
A	rs1031820	*COL11A1*	Lumbar low BMD	M + F	Tatars	5.495	2.30	1.130–4.675	0.019

Note: *p*-value, the value of the statistical significance level; χ^2^, Pearson’s chi-squared test; OR, odds ratio; CI, lower and upper limits of the 95% confidence interval.

**TABLE 5:** Identified associations by pairwise comparison in men and women separately.

Allele	Loci ID	Gene	Phenotype	Sex	Ethnos	χ^2^	OR	CI	*p*-value
A	rs10098470	*TPD52*	Osteoporosis	M	Total	10.150	5.12	1.682–15.560	0.001
A	rs10098470	*TPD52*	Osteoporosis	M	Russians	9.166	4.83	1.574–14.820	0.002
A	rs11540149	*VDR*	Osteoporosis	M	Total	11.810	2.18	1.386–3.436	0.001
A	rs11540149	*VDR*	Osteoporosis	M	Russians	11.960	2.27	1.412–3.642	0.001
G	rs1054204	*SPARC*	Osteoporosis	M	Total	7.213	1.42	1.099–1.836	0.007
G	rs1054204	*SPARC*	Osteoporosis	M	Russians	7.797	1.46	1.119–1.909	0.005
C	rs1712	*FBX05*	General fractures	M	Total	12.320	2.32	1.441–3.735	4.482e^−04^
A	rs10098470	*TPD52*	General fractures	M	Total	8.419	3.71	1.441–9.534	0.004
A	rs10098470	*TPD52*	General fractures	M	Russians	9.168	4.22	1.540–11.570	0.002
A	rs11540149	*VDR*	General fractures	M	Total	3.969	1.59	1.004–2.518	0.046
T	rs11614913	*miR-196a*	General fractures	M	Total	5.789	1.41	1.065–1.871	0.016
C	rs1712	*FBX05*	Typical fractures	M	Total	16.620	3.31	1.825–5.997	4.561e^−05^
A	rs10098470	*TPD52*	Typical fractures	M	Total	15.620	5.70	2.172–14.98	7.763e^−05^
A	rs10098470	*TPD52*	Typical fractures	F	Russians	5.118	5.46	1.051–28.370	0.024
A	rs11540149	*VDR*	Typical fractures	M	Total	6.877	1.99	1.180–3.361	0.009
T	rs1061947	*COL1A1*	Typical fractures	M	Russians	4.335	1.57	1.024–2.415	0.037
A	rs11540149	*VDR*	Femoral neck fracture	M	Russians	7.140	3.77	1.332–10.69	0.008
A	rs11540149	*VDR*	Femoral neck fracture	M	Total	5.682	3.26	1.170–9.082	0.017
G	rs6854081	*FGF2*	Femoral neck fracture	M	Total	5.079	2.87	1.103–7.462	0.024
G	rs6854081	*FGF2*	Femoral neck fracture	F	Russians	5.399	2.27	1.119–4.596	0.020
G	rs6854081	*FGF2*	Femoral neck fracture	F	Total	4.167	1.91	1.017–3.575	0.041
G	rs1042673	*SOX9*	Femoral neck fracture	M	Total	4.216	2.51	1.013–6.226	0.040
C	rs1712	*FBX05*	Lumbar fractures	M	Total	14.930	3.41	1.786–6.502	1.118e^−04^
A	rs10098470	*TPD52*	Lumbar fractures	M	Total	12.580	5.39	1.915–15.180	3.902e^−04^
A	rs10098470	*TPD52*	Lumbar fractures	F	Russians	8.980	11.29	1.550–82.170	0.003
A	rs11540149	*VDR*	Lumbar fractures	F	Total	5.536	2.37	1.132–4.950	0.019
A	rs11540149	*VDR*	Lumbar fractures	F	Russians	6.982	3.01	1.283–7.052	0.008
A	rs11540149	*VDR*	Lumbar fractures	M	Total	5.029	1.93	1.077–3.464	0.025
G	rs1031820	*COL11A1*	Lumbar fractures	M	Total	8.859	2.37	1.323–4.249	0.003
G	rs1042673	*SOX9*	Lumbar fractures	F	Tatar	7.875	7.00	1.487–32.94	0.005
C	rs1712	*FBX05*	Radial fractures	M	Total	7.711	6.33	1.452–27.590	0.006
A	rs11540149	*VDR*	Radial fractures	M	Russians	7.503	3.02	1.321–6.897	0.006
A	rs11540149	*VDR*	Radial fractures	M	Total	6.647	2.67	1.232–5.792	0.010
A	rs10793442	*ZNF239*	Radial fractures	F	Tatar	4.166	2.22	1.017–4.852	0.041
C	rs1712	*FBX05*	Low BMD in general	M	Total	3.905	1.57	1.002–2.460	0.048
A	rs11540149	*VDR*	Low BMD in general	M	Russians	7.872	2.16	1.248–3.742	0.005
A	rs11540149	*VDR*	Low BMD in general	M	Total	6.871	1.94	1.173–3.211	0.009
G	rs10098470	*TPD52*	Femoral neck low BMD	M	Total	4.215	3.09	0.997–9.591	0.040
G	rs9659030	*COL11A1*	Femoral neck low BMD	M	Total	4.361	1.66	1.028–2.664	0.037
G	rs1054204	*SPARC*	Femoral neck low BMD	M	Total	4.235	1.34	1.014–1.776	0.040
G	rs1054204	*SPARC*	Femoral neck low BMD	M	Russians	7.457	1.52	1.124–2.042	0.006
T	rs5854	*MMP1*	Femoral neck low BMD	M	Russians	4.467	1.41	1.025–1.943	0.035
A	rs11540149	*VDR*	Lumbar low BMD	M	Russians	4.222	1.76	1.021–3.038	0.040
G	rs1054204	*SPARC*	Lumbar low BMD	M	Russians	3.848	1.35	1.000–1.811	0.040
G	rs2910164	*miR-146a*	Lumbar low BMD	F	Russians	5.141	1.51	1.056–2.150	0.023
A	rs1042673	*SOX9*	Lumbar low BMD	F	Russians	5.141	1.61	1.065–2.421	0.023

In the total sampling, the highest number of associations was found for loci rs10098470 (*TPD52*) and rs11540149 (*VDR*), which are associated with both fractures and low BMD alone and in combination with a high level of statistical significance (Table 4).

The association of T locus rs1164913 (*miR-196a*), A locus rs11540149 (*VDR*), and A locus rs10098470 (*TPD52*) alleles with OP was found in a pooled sampling of men and women. However, when the cohorts of men and women were analyzed separately, the identified associations persisted only in men, except for rs1164913, and there was a new association of the G allele of locus rs1054204 (*SPARC*) with OP in general in men. When the sample was further stratified into subgroups based on the presence of fractures and low BMD levels separately, the rs1164913 locus was associated with general fractures and the rs1054204 locus was associated with low femoral neck BMD in men (Table 5). This indicates that primary OP is a genetically heterogeneous disease with different contributions of risky marker variants according to the sex.

This is also true for ethnicity, when cohorts are more homogeneous due to belonging to the same ethnic group, the association becomes more significant. For example, the A allele of the rs11540149 locus (*VDR*) showed an association with fractures in the pooled sampling of men and women overall (OR = 5.2), in Russians (OR = 7.24), and with typical fractures–OR = 6.85 (overall) and OR = 8.20 (in Russians).

When dividing the sampling by sex, we observed the persistence of the identified associations mainly in men despite the fact that their effect was lower than in women (Table 4). For example, the OR for the association of the C allele of the rs1712 locus (*FBX05*) with fractures overall in men reached 2.32 compared with 1.4 in the pooled sampling of men and women. However, no statistically significant associations were found with general fractures in women. The C allele of the rs1712 locus (*FBX05*) was associated with general fractures, as well as typical osteoporotic fractures and radial fractures, with OR increasing from 1.34 to 1.74, respectively.

**FIGURE 1:**
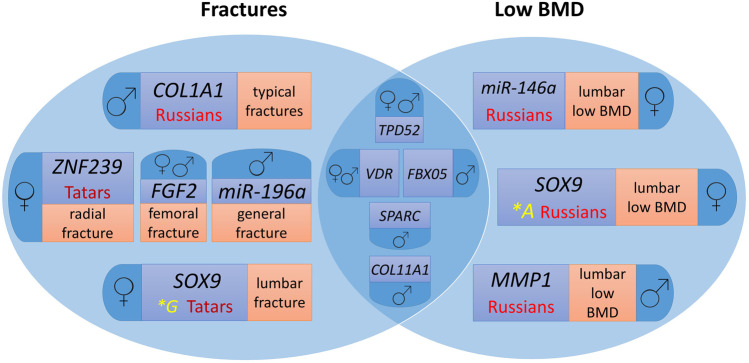
Genes involved in the formation of osteoporotic fractures and low BMD in men and women from the Volga-Ural region of Russia, taking into account ethnic characteristics. For easier perception, each phenotype localisation and ethnic specificity characteristics are highlighted in different colour.

Figure 1 presents a Venn diagram showing the contribution of the studied polymorphic variants of microRNA binding sites in target genes to fracture risk and low BMD individually and common markers for the two endophenotypes of primary OP by ethnicity and sex.

Polymorphic variants of the *VDR* and *TPD52* genes are common risk markers of fractures and low BMD for men and women, *FBX05* and *COL11A1* – for men. At the same time, the *SOX9*, *MMP1*, *ZNF239*, *COL1A1*, *FGF2*, *miR-146a*, and *miR-196a* gene loci are characterized by associations for separate groups depending on sex and ethnicity, as well as the localization of the pathological process (Figure 1).

In the total sampling of men and women, the association of the polymorphic variant rs10098470 of the *TPD52* gene with lumbar fractures in Russians (χ^2^ = 23.600, OR = 7.77, *p* = 1.183^e−06^) reaches the highest level of statistical significance (Table 3), which is also associated with a low level of BMD in general, as well as femoral neck in the pooled sampling, but with a lower level of significance. When the sampling was divided by sex, the associations with lumbar fractures remained in cohorts of men and women of Russian ethnicity.

In the sampling of women, variant A of the *SOX9* gene locus rs1042673, associated with lumbar fractures in women of Tatar ethnicity, has the highest odds ratio (OR = 7.00). At the same time, the G allele is associated with low BMD in women of Russian origin with a significantly lower odds ratio (OR = 1.61). The risk allele A of the locus rs10793442 of the *ZNF239* gene, which is probably a specific marker of radial fractures in women of Tatar ethnicity, reaches statistical significance.

In women, allele G of locus rs6854081 of the *FGF2* gene is associated with femoral neck fractures, with OR increasing from 4.167 to 5.399 in women of Russian ethnicity. Probably, there are population differences in the distribution of allele frequencies, which confirms the relevance of the search for ethnospecific markers of primary OP.

The loci associated with OP traits in men and women separately contribute to the notion of sex differences in the mechanisms of fracture risk and low BMD. The results require validation in independent samplings.

### 3.1 Assessment of sampling heterogeneity using meta-analysis tools

Due to ethnic heterogeneity and the presence of sexual dimorphism in OP, the authors assessed the genetic heterogeneity of the studied sampling using meta-analysis tools based on the PLINK software. Meta-analysis provides the optimal opportunity to find effects that are specific to the whole cohort. Heterogeneity of information (inherited genetic background, covariation of genotypes and phenotypes) affects not only statistical power but also increases the potential for false positives (Brien et al., 2018). Fixed-effects models assume that there is a common true genetic effect in all association studies, and any variation is explained by random error; on the other hand, random-effects models assume that there are different effect sizes in the association tests, and any differences, i.e., heterogeneity, are due to real population differences. This allows cohort information to be harmonized as much as possible. A pooled sampling of men and women of Russian and Tatar ethnicity was assessed, and then, men and women were assessed separately.

For the rs10098470 (*TPD52*) and rs11540149 (*VDR*) loci, the authors found high heterogeneity in the pooled sampling of men and women (I^2^>50%) and applied randomized *p* values (p(R)) for the random-effects model, which did not show the significance of these loci for low-BMD and fracture risk in the pooled sampling, general fractures, without regard to the BMD level. For the rs10098470 (*TPD52*) locus, heterogeneity is apparently due to the fact that the A allele is a marker of fractures and low BMD in combination and separately in Russians in general and for men and women separately, and the associations with fractures and low BMD of specific skeletal sites did not always coincide. The same patterns are characteristic of the A allele of the rs11540149 locus (*VDR*), which is associated with general fractures and standard localizations mainly in men, except for lumbar fractures and a low level of lumbar BMD in women, all associations were stronger in representatives of Russian ethnicity.

In the low-BMD sample, meta-analysis revealed a high level of heterogeneity for loci rs1031820, rs10793442, and rs1042673 (I^2^ = 73.04, 54.24, and 63.08, respectively). The G allele rs1031820 of the *COL11A1* gene was associated with lumbar fractures in the pooled sampling of men and men of Russian ethnicity, and the A allele was associated with low femoral neck BMD in the pooled group of men and women of Tatar origin due to differences in the frequency of the minor allele A, which was 3.5 times higher in men than in the sampling of women (0.301 and 0.084, respectively). The high level of heterogeneity at the locus rs10793442 of the *ZNF239* gene can probably be explained by the association with BMD only in Russians. For the *SOX9* gene rs1042673 locus, heterogeneity is observed in a sampling of women; this locus is associated with a low level of BMD in women of Russian origin. However, the risky allele in this case is allele A, whereas allele G is associated with lumbar fractures in women of Tatar ethnicity. It is necessary to expand the sample to confirm the obtained associations, as well as to replicate them in an independent sampling.

Thus, heterogeneity in ethnicity, sex, and fracture-associated and low BMD of individual localizations for loci rs10098470 (*TPD52*) and rs11540149 (*VDR*) in the studied cohorts of women and men from the Volga-Ural region was revealed, where the majority of the sampling consisted of Russians, belonging to the Slavic group, and Tatars, a Turkic group of the Altai language family, having diversity of ethnogenesis, diet and religion, but living on a common territory for a long time.

### 3.2 Prognostic models of fractures and low BMD levels

The conducted studies yielded molecular genetic risk markers for the development of OP in general, as well as its various phenotypes. However, the analysis of individual risk factors does not take into account their interaction, which is necessary for predicting the risk of developing the studied pathology. It is also of interest to evaluate the joint influence of genetic and clinical factors, such as body mass index (BMI) and BMD levels of the lumbar spine and femoral neck. In order to study the mutual influence of clinical and molecular genetic factors on the risk of OP and to create diagnostic algorithms, the authors performed statistical processing of the obtained results by logistic regression and ROC analysis methods with the calculation of the area under the curve (AUC), the values of which range from 0.6 to 0.7 indicating the average diagnostic value of the model; 0.7–0.8–good; 0.8–0.9–very good.

**FIGURE 2:**
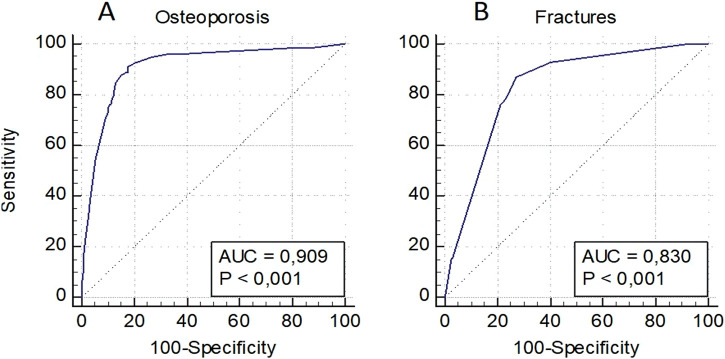
Prognostic models for assessing the risk of osteoporosis in general **(A)** and osteoporotic fractures **(B)** in women.

Predictive models were developed to assess the risk of OP in general, as well as osteoporotic fractures in women and men individually, and in a pooled sampling. The model for assessing the risk of OP in women included a low BMD in standard localizations, as well as loci rs6854081 (*FGF2*) and rs1054204 (*SPARC*); the model for predicting osteoporotic fractures included a low BMD and locus rs1712 of the *FBXO5* gene (Figure 2). The models were characterized by the highest level of predictive significance (AUC = 0.909 and AUC = 0.830, respectively). It should be noted that the exclusion of genetic loci from the analysis reduced the predictive value of the models (AUC = 0.844 and AUC = 0.805, respectively).

**FIGURE 3:**
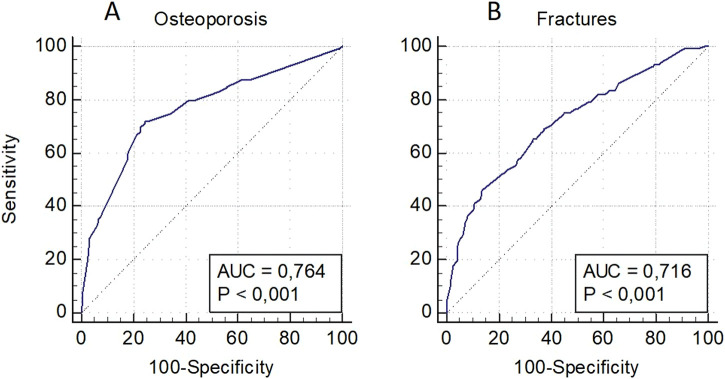
Prognostic models for osteoporosis risk assessment in general **(A)** and osteoporotic fractures **(B)** in men.

The model for OP risk assessment in men included a decreased level of BMD in standard localizations and the locus rs1712 (*FBXO5*) and rs11540149 (*VDR*), while the model for fractures included a low level of BMD and the loci rs1712 (*FBXO5*), rs1042673 (*SOX9*), and rs10793442 of the *ZNF239* gene (Figure 3). The models were characterized by a sufficient level of predictive significance (AUC = 0.764 and AUC = 0.716, respectively), there was a tendency of decreasing predictive value when genetic predictors were excluded (AUC = 0.740 and AUC = 0.649, respectively).

**FIGURE 4:**
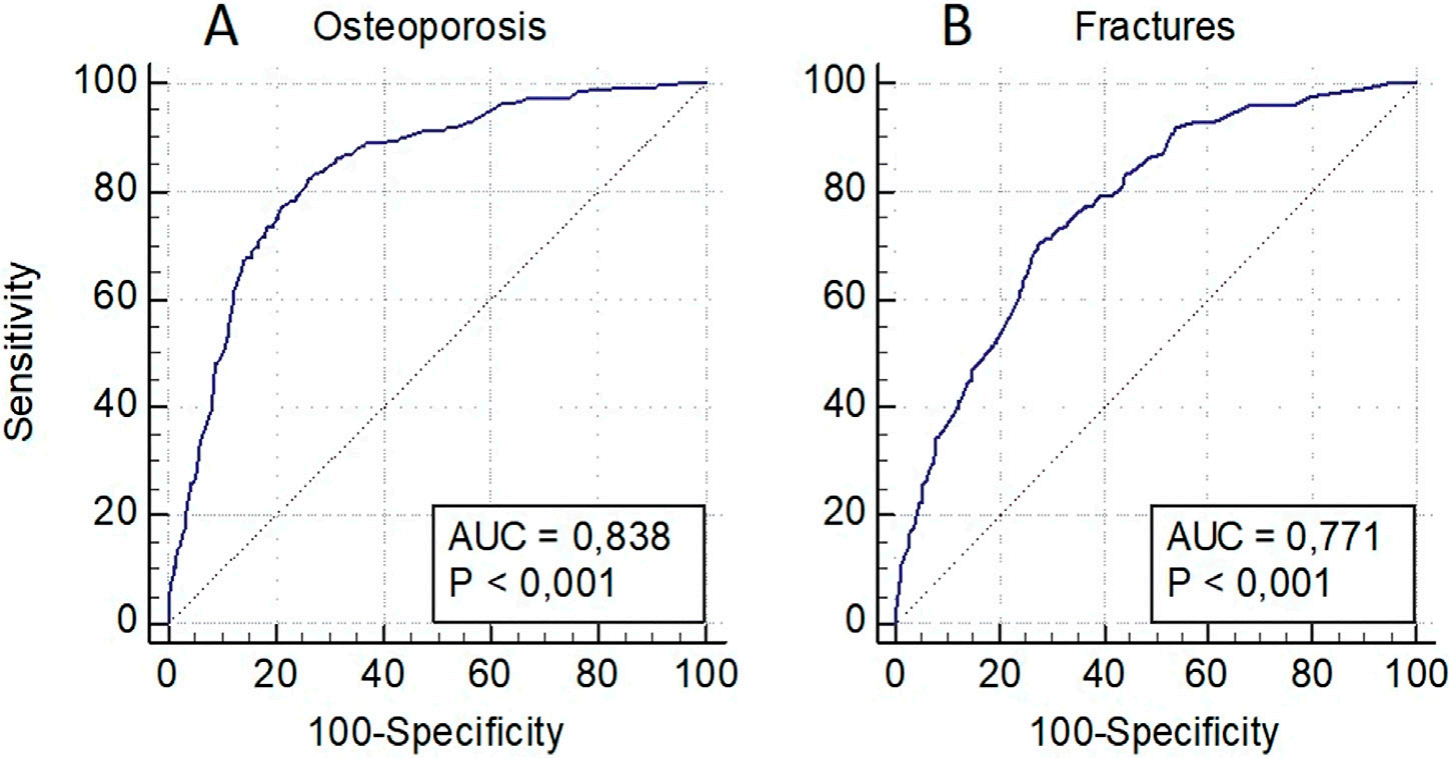
Predictive models for assessing the risk of osteoporosis in general **(A)** and of osteoporotic fractures **(B)** in a pooled sampling of men and women.

The OP risk assessment model in the pooled sampling of men and women also included decreased BMD levels in standard localizations, as well as loci rs1712 (*FBXO5*), rs6854081 (*FGF2*), rs1054204 (*SPARC*), rs10793442 (*ZNF239*), and the model for fractures–decreased BMD levels and loci rs1712 (*FBXO5*), rs10793442 (*ZNF239*), and rs11614913 of the *miR-196a* gene (Figure 4). The models were characterized by a sufficient level of predictive significance (AUC = 0.838 and AUC = 0.771, respectively), the trend of decreasing predictive value when genetic predictors were excluded was maintained (AUC = 0.808 and AUC = 0.720, respectively).

Thus, the conducted logistic regression and ROC analyses made it possible to identify several models with high prognostic significance for predicting the risk of primary OP and osteoporotic fractures in men and women in general and separately, which confirmed the role of microRNA binding site loci in the *FBXO5*, *FGF2*, *SPARC*, *ZNF239*, and *miR-196a* genes in the formation of the disease phenotype obtained by association analysis as well. The loci rs10098470 (*TPD52*) and rs11540149 (*VDR*), for which high heterogeneity was detected, were not included in the models with high statistical significance.

### 3.3 Prediction of mRNA-microRNA interactions depending on binding site changes in the 3′UTR of mRNAs of *FGF2, TPD52* and *FBXO5* genes

Based on the association study data of microRNA binding site polymorphic variants rs6854081, rs10098470 and rs1712, which showed the highest association signal with osteoporosis phenotypes, we performed a comparative analysis of predicted microRNAs with potential affinity for mRNA of *FGF2, TPD52* and *FBXO5* genes. It was done to test how binding site variation at the studied loci affected affinity for microRNAs, whether the qualitative composition of predicted microRNAs changed depending on the presence of the alternative allele, and whether the localization of microRNA binding to mRNA changed in the 3′UTR region of mRNA.

We found that *in silico*, at risk allele C, a novel microRNA hsa-miR-6838-3p was predicted in transcript 002,006.6, which had no affinity for the reference mRNA sequence. Through analysis of the entire mRNA transcript sequence, we found that a new binding site in the 3′UTR, which previously had no affinity for the reference sequence, was predicted for the microRNAs hsa-miR-34a-5p, hsa-miR-380-3p, hsa-miR-654-3p, hsa-miR-3649, and hsa-miR-6770-3p (Table 6).

**TABLE 6:** Predicted microRNAs, their binding free energy, and binding site localization depending on the polymorphic variant rs6854081 in the mRNA transcript of *FGF2* gene 002,006.6 at alternative C allele of *FGF2*.

Acquired affinity for a novel microRNA			Changing the binding position with mRNA for target microRNAs		
microRNA	Binding energy	Binding site	microRNA	Binding energy	Binding site
hsa-miR-6838-3p	−8.72	3′UTR	hsa-miR-34a-5p	−9.05	3′UTR
			hsa-miR-380-3p	−8.24	3′UTR
			hsa-miR-556-3p	−9.25	Up to 3′UTR
			hsa-miR-6770-3p	−10.14	3′UTR
			hsa-miR-654-3p	−10.63	3′UTR
			hsa-miR-3649	−9.93	3′UTR

It was revealed that at risk allele C in the 3′UTR of *TPD52* gene in transcript NM_001287144.2, three microRNAs lost affinity to the reference sequence: hsa-miR-548d-3p, hsa-miR-873-3p, hsa-miR-1537-3p. The microRNA hsa-miR-6753-3p is also predicted to have affinity for the 3′UTR mRNA with high binding energy (−13.49) (Table 7). Analysis of predicted transcript interactions with NM_005079.4 microRNAs showed that these microRNAs retained affinity for mRNA, but the binding site changed to a position upstream of the 3′UTR region. Analysis of transcript NM_001025253.3 in the 3′UTR predicts the appearance of hsa-miR-6753-3p microRNA affinity.

**TABLE 7:** Predicted microRNAs, their binding free energy, and binding site localization depending on the polymorphic variant rs10098470 in the mRNA transcript of the *TPD52* gene NM_001287144.2 at alternative allele C of the *TPD52* gene.

Lost affinity for microRNAs			Changing the binding position with mRNA for target microRNAs		
microRNA	Binding energy	Binding site	microRNA	Binding energy	Binding site
hsa-miR-548d-3p	−8.03	3′UTR	hsa-miR-6753-3p	−13.49	3′UTR
hsa-miR-873-3p	−8.17	3′UTR			
hsa-miR-1537-3p	−8.04	3′UTR			

Table 8 shows the results of comparison of all predicted microRNAs, which were filtered based on the appearance or loss of affinity for mRNA or on the change of the binding site in the 3′UTR of the *FBXO5* gene. Comparative analysis of the predicted microRNAs for transcript NM_012177.5 revealed several changes. At risk allele A, affinity for the new microRNAs hsa-miR-105-5p, hsa-miR-379-5p, hsa-miR-552-3p, and hsa-miR-617 appeared, and affinity for hsa-miR-187-5p, hsa-miR-196b-3p, and hsa-miR-1273c was completely lost (Table 8). In addition, a change in the microRNA binding site with localization in the 3′UTR with high binding energy was detected for microRNAs hsa-miR-139-5p, hsa-miR-3125, hsa-miR-122b-5p and hsa-miR-5579-3p.

**TABLE 8:** Predicted microRNAs, their binding free energy, and binding site localization depending on the rs1712 polymorphic variant in *FBXO5* gene mRNA transcript NM_012177.5 at alternative C allele of *FBXO5.*

Acquired affinity for microRNA			Lost affinity for microRNAs		
microRNA	Binding energy	Binding site	microRNA	Binding energy	Binding site
hsa-miR-105-5p	−8.07	3′UTR	hsa-miR-187-5p	−15.85	3′UTR
hsa-miR-379-5p	−8.09	3′UTR	hsa-miR-196b-3p	−8.42	3′UTR
hsa-miR-552-3p	−8.8	3′UTR	hsa-miR-1273c	−8.99	3′UTR
hsa-miR-617	−8.12	3′UTR			

Comparative analysis of predicted microRNAs for transcript NM_001142522.3 revealed that the risk allele resulted in a new affinity for the microRNAs hsa-miR-105-5p, hsa-miR-379-5p, hsa-miR-552-3p, hsa-miR-617, and hsa-miR-3085-5p, and loss of affinity for hsa-miR-187-5p, hsa-miR-382-3p, hsa-miR-196b-3p, hsa-miR-549a-3p, hsa-miR-2277-3p, and hsa-miR-1273c (Table 9).

**TABLE 9:** Predicted microRNAs, their binding free energy, and binding site localization as a function of the rs1712 polymorphic variant in the mRNA transcript of the *FBXO5* NM_001142522.3 gene at alternative C allele of *FBXO5*.

Acquired affinity for microRNA			Lost affinity for microRNAs		
microRNA	Binding energy	Binding site	microRNA	Binding energy	Binding site
hsa-miR-105-5p	−8.07	3′UTR	hsa-miR-187-5p	−15.85	3′UTR
hsa-miR-379-5p	−8.09	3′UTR	hsa-miR-382-3p	−10.24	3′UTR
hsa-miR-552-3p	−8.8	3′UTR	hsa-miR-196b-3p	−8.42	3′UTR
hsa-miR-617	−8.12	3′UTR	hsa-miR-549a-3p	−11.18	3′UTR
hsa-miR-3085-5p	−9.54	3′UTR	hsa-miR-2277-3p	−9.68	3′UTR
			hsa-miR-1273c	−8.99	3′UTR

In addition, changes in the localization of binding sites were noted. Thus, for microRNAs hsa-miR-139-5p, hsa-miR-6878-5p, hsa-miR-3125, hsa-miR-3127-5p, and hsa-miR-3945, the binding site moved to the 3′UTR-region of the gene with high binding energy. At the same time, the microRNA binding sites hsa-miR-146b-5p, hsa-miR-181d-3p, hsa-miR-498-3p, hsa-miR-371b-5p, and hsa-miR-5194 binding site moved upstream beyond the 3′UTR (Table 10).

**TABLE 10:** Predicted microRNAs, their binding free energy, and binding site localization as a function of the rs1712 polymorphic variant in the mRNA transcript of the *FBXO5* NM_001142522.3 gene at the alternative C allele of *FBXO5*.

Changing mRNA binding position for target microRNAs					
microRNA	Binding energy	Binding site	microRNA	Binding energy	Binding site
hsa-miR-139-5p	−12.49	3′UTR	hsa-miR-146b-5p	−8.21	Up to 3′UTR
hsa-miR-6878-5p	−10.94	3′UTR	hsa-miR-181d-3p	−10.23	Up to 3′UTR
hsa-miR-3125	−14.84	3′UTR	hsa-miR-498-3p	−9.91	Up to 3′UTR
hsa-miR-3127-5p	−11.31	3′UTR	hsa-miR-371b-5p	−11.05	Up to 3′UTR
hsa-miR-3945	−13.34	3′UTR	hsa-miR-5194	−15.43	Up to 3′UTR

The point is that an alternative allele of the binding site can lead to a change in the composition of microRNAs with potential affinity for mRNA. In turn, a change in the composition of microRNAs can lead to a functional change in protein production activity and play a biological role in the dysregulation of the activity of genes involved in bone tissue metabolism and homeostasis.

## 4 Discussion

Primary OP is a multifactorial and genetically heterogeneous disease with complex etiopathogenesis. Hereditary factors are involved in the formation of the disease, which have a pronounced sex and ethnic heterogeneous component, significantly complicating the search for molecular genetic predictors of OP with high significance and protocols for the diagnosis of the disease using DNA markers. Identification of such markers would allow the specialists not only to develop ways of early diagnosis of fractures, but also to reveal complex issues of basic research on the molecular pathogenesis of the disease, taking into account the diversity of ethnic groups of the world’s populations. To date, these tasks remain largely unrealized, and the problem of genetics and epigenetics of OP in certain regions of the world with a diverse genetic landscape of the gene pool of indigenous peoples remains understudied. A new promising direction in the field of basic research on primary osteoporosis is the search for disease markers among polymorphic variants of microRNA binding sites in mRNA genes, as well as functional studies of the role of these polymorphisms, due to the fact that these data are of great value for the development of targeted RNA therapy for osteoporosis and bone tissue diseases in general.

Each microRNA has certain chemical affinity, which is affinity for the target mRNA in the regulation of which it is involved. The efficiency of their interaction is determined by different thermodynamic and chemical properties of these macromolecules. The affinity of mRNA and microRNA affects the regulation of gene expression–the higher it is, the stronger is the effect of RNA-induced gene silencing (RNA interference), i.e., suppression of gene expression (up to complete degradation of mRNA with cessation of translated protein expression) (Brien et al., 2018).

Several factors are known to influence the affinity of mRNAs and microRNAs:

1. Complementarity of the canonical interaction between the “seed”-region of the microRNA (2–8 nucleotides at the 5′-end) and the 3′-untranslated region (UTR) in the mRNA binding site of the target gene. The higher is this complementarity, the more effective is the RISC complex of RNA interference (or discrete microRNA without the RISC complex) (Bartel, 2009).
2. Non-canonical interaction of the “seed”-region of microRNA with the coding (CDS) and 5′-UTR regions of mRNA. According to CLASH data, most of the interactions (about 58%) lie in the CDS region, 38% lie in the 3′-UTR, and 4% are in the 5′-UTR in mRNA. However, non-canonical interactions are not as well studied as in the case of microRNA interactions with the 3′-UTR of mRNAs. Besides, their functional significance in mRNA upregulation was not established. Therefore, their role in influencing the regulation of gene expression needs additional functional studies (Helwak et al., 2013).
3. Spatial configuration of the secondary structure of mRNA (the double helix of RNA is formed by two linear molecules connected to each other along the entire length by hydrogen bonds). It is known that complex conformational changes in the spatial orientation of mRNA molecules affect the efficiency of interaction between mRNA and microRNAs that have affinity for binding sites in different regions of mRNA (Yang et al., 2020).

Moreover, SNPs of binding sites can affect the secondary structure of mRNA, potentially limiting the accessibility of the microRNA binding site to the binding centers of the RNA-induced gene silencing complex, thereby, affecting both the composition of microRNAs and their spatial accessibility for binding (Rykova et al., 2022).

The field of research on the influence of polymorphisms in 3′UTR-regions of mRNA on the risk of various diseases is actively developing. However, due to the huge number of microRNA variations in humans (more than 2,500), as well as the huge representation of microRNA binding sites in the genome, and taking into account the presence of multiple mRNA transcripts for each gene, an unprecedented number of possible and complex variants of functional consequences of microRNA-mRNA interactions appears. To reveal the mechanisms and significance of these interactions, there are various bioinformatic tools that aim to establish the role of aberrations of these interactions in the risk of disease development, including osteoporosis. Therefore, an urgent task is to search for the most functionally significant changes in predicted mRNA-microRNA interactions and their role in disease pathogenesis depending on polymorphic variants of microRNA binding sites, for which a statistically significant association with pathology phenotypes were shown in the studied patient cohorts (Chhichholiya et al., 2021).

### 4.1 FGF2

Fibroblast growth factor type 2 (*FGF2*) is a highly pleiotropic member of a large family of growth factors with a broad spectrum of activity, including proliferation and self-renewal of human pluripotent stem cells. The *FGF2* gene does not have alternative splicing (Nickle et al., 2023). Instead, isoforms of the *FGF2* protein are expressed from a single mRNA: a high molecular weight 34 kDa HMW isoform localized to the cell nucleus and a low molecular weight 18 kDa LMW isoform predominantly localized to the membrane as a ligand for fibroblast growth factor receptor (FGFR). HMW has an inhibitory effect on mineralization and LMW promotes bone formation (Coffin et al., 2018).

Since the role of aberrant HMW expression in low bone mineralization was proven, we focused on the transcript encoding the HMW isoform of *FGF2*. Using IntaRNA algorithms (Mann et al., 2017), we evaluated predictions of microRNA and NM_002006.6 (HMW mRNA) transcript interactions and found that the risk allele of the rs6854081 polymorphic variant in the 3′UTR *in silico* results in affinity for *FGF2* gene mRNA with a novel microRNA–hsa-miR-6838-3p.

Hsa-miR-6838-3p is overexpressed in patients with osteosarcoma (Wang et al., 2022). In addition, decreased levels of miR-6838-5p increase the level of *FGF2* gene expression (Zhang et al., 2023). This was found in a study by Zhang et al. (2023), who found that Circ_0001667 RNA was associated with breast cancer and angiogenesis processes involving miR-6838-5p. Hsa-miR-34a-5p with affinity for in the 3′UTR region of the mRNA is associated with the induction of osteogenic differentiation of bone marrow mesenchymal stem cells. This microRNA modulates bone metabolism by targeting HDAC1 and stimulating ER-α transcription (Sun et al., 2023). The interaction of miR-6838-3p and miR-34a-5p with *FGF2* gene mRNA may be associated with decreased *FGF2* expression; however, additional studies are needed to test this hypothesis.

In the study by Lei et al. (2011), rs6854081 (*FGF2*) is a functionally investigated locus. They not only found an association of this variant with low BMD, but also identified aberrant expression of the *FGF2* gene in women with osteoporosis, with the rs6854081 locus being associated with altered osteoclast differentiation (Lei et al., 2011). We reproduced a statistically significant association of this locus with OP: in our sample, the G allele of rs6854081 is a predictor in a prognostic model of the risk of low BMD formation and is also associated with femoral neck fractures in men and women. Studies show that the *FGF2* gene transcript with the major T allele of the rs6854081 locus has a lower expression level of monocytes and is associated with a high level of BMD, which only confirms the potentially risky role of the C allele.

### 4.2 TPD52

D52 is one of the members of the tumor protein family (Abe et al., 2021). Several studies identified the *TPD52* gene sequence by its increased expression in human breast cancer tissue and in lung cancer cell lines. Several studies identified overexpression of the *TPD52* gene in osteosarcoma cell lines (Abe et al., 2021). About 80% of the *TPD52* transcript was initially identified as a 3′-untranslated region, so a large number of microRNAs involved in the regulation of *TPD52* gene expression was identified (Han et al., 2015). This gene has 29 transcripts (splice variants), 208 orthologs, and 3 paralogs. Eight of them encode different protein isoforms. The polymorphic variant rs10098470 is localized in the *TPD52* gene encoding tumor protein D52. The function of this protein in the pathogenesis of osteoporosis was not studied. Nevertheless, the variant rs10098470 was still identified in the GWAS study with a high level of significance (Lei et al., 2011). Studies showed that TPD52 family proteins were not involved in osteoclast differentiation. Still, they indicated that the protein played an important role in terminal chondrocyte differentiation during endochondral ossification, maintaining the pre-hypertrophic state of chondrocytes (Ito et al., 2017). Hence, given the replicated association in two cohorts [ours and Lei et al. (2011)], and considering the important role of TPD52 protein in ossification, the rs10098470 variant deserves further functional studies. Using IntaRNA algorithms, we evaluated predictions of microRNA and NM_002006.6 transcript (HMW mRNA) interactions and found that the risk allele of the rs6854081 polymorphic variant in the 3′UTR *in silico* results in affinity for FGF2 gene mRNA with a novel microRNA–hsa-miR-6838-3p.

### 4.3 FBXO5

*FBXO5* is an F-box 5 protein that constitutes one of the four subunits of the ubiquitin protein-ligase complex called SCF (SKP1-cullin-F-box), functioning in a phosphorylation-dependent ubiquitination process. *FBXO5* gene expression is upregulated in many human cancers and is associated with chromosomal instability. A marked increase in mineralization was observed in groups overexpressing both transcripts compared to controls. Knockdown of *FBXO5* resulted in decreased migration and osteogenic differentiation functions. In contrast, overexpression of *FBXO5* enhanced the potential for migration and osteogenic differentiation. These data suggest that *FBXO5* plays a positive role in the regulation of migration and osteogenic differentiation (Liu et al., 2018). In the work of Lei et al. (2011), the most significant level of association with the level of femoral neck BMD in women was revealed for the rs1712 variant of the *FBX05* gene (*p* = 2.5e^−3^). We replicated this result. However, the difference is that in our work, a significant association was found only in men, who had the C allele of the rs1712 locus (*FBX05*) associated with fractures of all localizations except the femoral neck, as well as with a low level of BMD in general. In the PolymiRTS database, microRNA miR-549a has affinity for this site, which level is elevated in Huntington’s chorea and decreased in fibroblasts in patients with nonsyndromal cleft palate.

At present, the role of the *FBX05* gene in the pathogenesis of osteoporosis remains largely unknown, but the reproducibility and high significance in OP risk indicate the high prognostic potential of the rs1712 locus. We assessed whether the affinity for microRNAs of transcripts of this gene changes depending on the rs1712 *in silico* variant and found that the risk allele was associated with a significant change in microRNAs potentially interacting with the gene’s mRNA. Among the microRNAs for which a new affinity appeared, miR-617 is of interest, as this microRNA is involved in the regulation of osteogenic cell differentiation (Ge et al., 2020). Among the microRNAs that lost affinity, the most interesting is miR-382, which stimulates osteogenic differentiation in MG63 cells (Su et al., 2023). Further functional studies are needed.

Thus, within the framework of the study, for the first time, new regularities were revealed in the little-studied area of epigenetic factors of primary OP–the role of polymorphic variants of microRNA binding sites in the risk of fracture development and the formation of low BMD. The study of the influence of polymorphic variants of microRNA binding sites on the risk of OP is important because the discovered patterns suggest a high role of polymiRTS in increasing the risk of osteoporotic fractures, and therefore can be of potentially great value for the development of methods of early diagnosis of OP to prevent fractures.

## 5 Contribution of the study

The results of this study contribute to the understanding of epigenetically mediated mechanisms of fracture risk and low BMD in men and women. It was confirmed that polymorphic variants of microRNA binding sites in mRNAs of structural and regulatory genes of bone metabolism played an important role in the formation of the risk of primary OP.

## 6 Study limitations

Limitations of the study are the relatively small sampling size and the number of the studied polymorphic loci. In addition, the results were not replicated in other cohorts from Russia.

## 7 Conclusion

Through comprehensive and stratified analysis, the authors identified common and specific DNA markers of osteoporotic phenotypes. This study indicates that the variability of the BMD level and the formation of osteoporotic fractures are based on sex and localization specificity, which polymorphic variants of microRNA binding sites in the mRNA of bone metabolism genes are associated with. Sexual dimorphism is one of the most significant factors in the etiology of OP. Nevertheless, in primary forms of the disease with late manifestation, there are apparently common markers characteristic of postmenopausal women and age-matched men. Thus, new data on epigenetically mediated factors involved in the pathogenesis of OP in general, fractures and low BMD separately in women and men from the Volga-Ural region of Russia, taking into account their ethnicity, were obtained.

## Data Availability

The datasets presented in this study can be found in online repositories. The names of the repository/repositories and accession number(s) can be found below: https://datadryad.org/stash, DOI: 10.5061/dryad.gb5mkkx06.
